# Safety and Tolerability of Antipsychotic Drugs in Pediatric Patients: Data From a 1-Year Naturalistic Study

**DOI:** 10.3389/fpsyt.2020.00152

**Published:** 2020-03-24

**Authors:** Giuseppe Cicala, Maria A. Barbieri, Vincenza Santoro, Carmela Tata, Pia V. Colucci, Francesca Vanadia, Flavia Drago, Carmelita Russo, Paola M. Cutroneo, Antonella Gagliano, Edoardo Spina, Eva Germanò

**Affiliations:** ^1^Department of Clinical and Experimental Medicine, University of Messina, Messina, Italy; ^2^Childhood and Adolescence Neuropsychiatry, Azienda Sanitaria Provinciale 8, Syracuse, Italy; ^3^Complex Operative Unit of Neurology for Mental Retardation, IRCCS Oasi Maria SS, Enna, Italy; ^4^Childhood Neuropsychiatry, Arnas Civico di Cristina Benfratelli, Palermo, Italy; ^5^Childhood Neuropsychiatry, S. Marta and S. Venera Hospital, Azienda Sanitaria Provinciale 3, Catania, Italy; ^6^Regional Pharmacovigilance Center, Siciliy, AOU Policlinico G. Martino, Messina, Italy; ^7^Child and Adolescent Neuropsychiatry, Department of Biomedical Sciences, University of Cagliari and “G. Brotzu” Hospital Trust, Cagliari, Italy; ^8^Department of Adulthood and Developmental Age Human Pathology “Gaetano Barresi”, University of Messina, Messina, Italy

**Keywords:** antipsychotics, pediatric patients, long-term monitoring, naturalistic setting, adverse drug reaction (ADR), tolerability

## Abstract

**Background:** Antipsychotic drugs (APs) are increasingly used to treat a variety of psychiatric disorders in children and adolescents. However, their safety and tolerability profiles, when used in a developmental age context, show different characteristics from the ones observed in adult patients. Treatment with APs in pediatric patients is often long-term. However, the tolerability data regarding these patients mostly derive from short-term studies.

**Methods:** Starting from April 2017, for a 1-year period, patients between 4 and 18 years of age followed by five units of developmental age neuropsychiatry, who initiated a treatment with at least an AP (ATC class N05A) were included into the study. Patient-related data have been collected at baseline and regularly thereafter, as allowed by the clinical routine. Changes to continuous variables over time have been analyzed using a linear mixed model in subsamples of our population treated with risperidone or aripiprazole.

**Results:** During the observation period, 158 patients were initially enrolled, but only 116 completed 12 months of therapy with an AP. Risperidone was the most used AP (*n* = 52) followed by aripiprazole (*n* = 44) and olanzapine (*n* = 7). For both the aripiprazole and risperidone groups, the mean body mass index (BMI) (*P* < 0.001 for both groups) and heart rate (*P* = 0.026 for aripiprazole group and *P* < 0.001 for the risperidone one) values significantly increased over time. The mean prolactin concentration value significantly increased over time only in the risperidone group (*P* = 0.04). Eighty-six patients experienced at least one adverse drug reaction (ADR), accounting for a total of 238 specific reactions, with the most frequent being weight gain (*n* = 34), increased serum prolactin levels (*n* = 21), hyperphagia (*n* = 20), and hypercholesterolemia (*n* = 14). Among these, only 24 ADRs were classifiable as serious.

**Conclusions:** The results of this study confirm that risperidone and aripiprazole are relatively well-tolerated therapeutic options for the treatment of a variety of psychiatric disorders in pediatric patients. However, in findings such as statistically significant increments of BMI and heart rate mean values, the variations over time in prolactin levels observed with risperidone and the differences between the two drugs remark the necessity of systematic monitoring.

## Introduction

Antipsychotic drugs (APs) are the mainstay of treatment for schizophrenia. This class of medications can be divided into two groups, those referred to as typical or first-generation APs (FGAs) and the more recently developed agents, known as atypical or second-generation APs (SGAs) (1). Several improvements have been made to the pharmacodynamic profile of these drugs during the development of the SGAs, such as the lower dopamine receptor occupancy and the blockage of serotonin receptors in the cortical limbic pathways. These modifications reduced the incidence of important adverse drug reactions (ADRs) such as the excessive sedation and extrapyramidal symptoms contributing to a higher perceived safety over the FGAs (2). This is one of the most important factors that has led to a substantial increase in the use of APs, especially of the SGAs, over the past two decades, to treat children and adolescent patients in most developed countries (3, 4). Today, APs are an important therapeutic option in the treatment of schizophrenia as well as of a variety of disorders such as aggression and irritability secondary to autism spectrum disorders or intellectual disabilities, tics or Tourette disorders, and mood and conduct disorders (5–7). The growing use of these agents has also been associated with an extensive off-label use (8–10) caused by the lack of premarketing registration studies and, by consequence, of approved use cases for APs in pediatric patients. Moreover, the efficacy and safety of APs when used in this particular population are still supported by few studies often plagued by several methodological limitations such as a short observational period, a relatively low number of involved subjects, and the exclusion of patients (such as those presenting comorbidities or being treated with more than one drug) that will eventually be treated with APs. Young patients affected by bipolar disorder, psychosis, or schizophrenia have been shown to be particularly prone to experiencing ADRs due to APs (11). Also, children and adolescents seem to present a higher sensitivity to several ADRs such as neurologic disturbances, hyperprolactinemia, cardiovascular-related adverse events, weight gain, and related metabolic features in comparison to adult patients (12–14). These are consequences of the physiological variations typical of the developmental age, which could interfere with both the pharmacodynamic and pharmacokinetic profiles of the APs (15). Antipsychotic drugs also differ from each other across a wide range of pharmacokinetic and pharmacodynamic properties and by consequence in their safety and tolerability profiles (14, 16). Taking as an example the metabolic-related ADRs, the highest risk of weight gain in pediatric patients seems to be associated with the administration of olanzapine and clozapine followed by risperidone, quetiapine, haloperidol, aripiprazole, and ziprasidone. On the other hand, ziprasidone and clozapine are more commonly associated with the onset of cardiovascular adverse events, such as electrocardiogram (ECG) abnormalities and hypertension (17, 18). As far as hyperprolactinemia is concerned, risperidone seems to induce it quite frequently, whereas aripiprazole does not seem to show any effects on prolactin (PRL) levels (14, 16). Treatment with APs in pediatric patients is often long-term (19). However, a chronic exposure to APs during the developmental age could potentially lead to dysfunctional modifications of both metabolic and neurological processes, for example, a prolonged hyperprolactinemia can cause a delay in pubertal maturation, menstrual disturbances, gynecomastia, and sexual dysfunction, making it far more distressing for an adolescent than for an adult subject (20). Indeed, to date, the safety of these medications for long-term treatments in pediatric patients remains a subject of debate, and several important safety concerns previously highlighted in literature are still not yet resolved (13, 15, 21–23). Therefore, it is clear that close and long-term monitoring of pediatric patients treated with APs in a real-world setting could be useful to clinicians in the process of tailoring the therapies for the single patient and for the identification and treatment of therapy-related conditions (e.g., metabolic dysregulation) as previously stated by other authors (24). In addition to that, such monitoring could be helpful for a better definition of the safety and tolerability profiles of these drugs especially in the long term, as highlighted by previous studies based in a real-world setting (25). For these reasons, we carried out a 1-year naturalistic study involving pediatric patients treated with APs. We evaluated the changes in the main clinical, cardiovascular, and laboratory parameters that according to the guidelines published by the National Institute for Clinical Excellence (NICE) (26) should be monitored regularly and systematically throughout treatment with antipsychotic medication. These observations were integrated with a descriptive analysis of identified ADRs.

## Methods

### Study Design

This observational prospective cohort study was conducted as part of the operational framework of an active pharmacovigilance project focused on the evaluation of the safety and tolerability of APs in children and adolescents. The study was notified to and then approved by the ethical committee of the coordinating center, the University Hospital “AOU Policlinico G. Martino” of Messina on August 1, 2016. Five neuropsychiatry units specialized in diagnosis and treatment of psychiatric disorders in children and adolescents took part in the project coordinated by a unit of clinical pharmacology. In the period from April 2017 to April 2018, pediatric patients (aged ≤ 18 years), both inpatients and outpatients, who started treatment with any antipsychotic agent within the participating centers were included in the study. No restrictions were applied in terms of sex, type of psychiatric condition affecting the patient, and use of concomitant drugs. Patients were enrolled only after their parents or guardians, informed about the methods and aims of the project, agreed for them to participate by signing an informed consent form. After the treatment initiation, follow-up visits were planned according to the normal clinical routine. During the follow-up visits, the patients were monitored performing neuropsychiatric examinations, ECG, blood tests, and, if necessary, a pharmacological reevaluation (in terms of dose adjustment, discontinuation, or switch to another drug). Moreover, the assessment of eventual ADR(s) that occurred during the therapy was also performed.

### Data Collection

The clinicians involved into the study were supplied with a standardized electronic form, divided into sections corresponding to the different type of data recorded, and proceeded with the data collection. Once collected in anonymous form, the data were sent to the coordinating center, the participating staff of which took care of transferring them into a custom database developed using Microsoft Access®. In order to establish a comprehensive baseline, at the time of treatment initiation, data on patient's characteristics such as the type of patient service (inpatient or outpatient), age, sex, and psychiatric diagnosis were collected. The primary psychiatric diagnoses were coded based on the fifth edition of the *Diagnostic and Statistical Manual of Mental Disorders* (*DSM-5*) and the 10th revision of the *International Statistical Classification of Diseases and Related Health Problems* (*ICD-10*). Moreover, information regarding the AP(s) administered, administration route, dosage, and treatment duration was also recorded. Given the observational nature of the study, no limitations were imposed on clinicians concerning eventual dose adjustments or therapy discontinuation. Data collected from patient examination such as height, body mass index (BMI), systolic and diastolic blood pressure, heart rate and values of the mean QT interval, and concentrations of fasting glucose, cholesterol, high- and low-density lipoproteins, triglycerides, PRL, free triiodothyronine, free thyroxine, thyroid-stimulating hormone, and white blood cells and the neutrophils percentage were all recorded. Once the baseline was established, the data collection continued regularly thereafter concurrently with the follow-up visits. If during the therapy clinicians observed an adverse event possibly related to the AP administration, a description of the ADR, including symptoms, date of occurrence, seriousness, and outcome, was recorded into a dedicated section of the monitoring form. An ADR was classified as serious if it was definable as an untoward medical occurrence that resulted in death, was life-threatening, required inpatient hospitalization or prolongation of existing hospitalization, resulted in persistent or significant disability or incapacity, or resulted in a congenital anomaly/birth defect or clinically relevant conditions based on the Important Medical Event list (27) or on clinical judgments, all of this in accordance to what was stated in the International Conference on Harmonization of Technical Requirements for Registration of Pharmaceuticals for Human Use, clinical safety data management: definitions and standards for expedited reporting E2A guideline (28). The ADR outcomes were classified into four categories: fully recovered, improved, not resolved, and worsened. The reported ADRs were coded using the Medical Dictionary for Regulatory Activities hierarchical classification at preferred term (PT) level and grouped at system organ class (SOC) level. In accordance to the current European legislation on pharmacovigilance, clinicians compiled the appropriate ADR reporting form made available by the Italian Medicine Agency (Agenzia Italiana del Farmaco [AIFA]) and sent that to a qualified person responsible for pharmacovigilance of their respective health structures who took care of the ADR report uploading into the Italian Pharmacovigilance Network (Rete Nazionale di Farmacovigilanza). Of note, only the clinicians, without any solicitation, made the decision to report the suspected ADRs. The strength of the relationship between the suspected drug therapy and the onset of an ADR was established by applying the Naranjo algorithm (29). The ADRs were recorded during the entire observation period for all the patients enrolled as long as they were in treatment with an AP independently from the treatment duration or the eventual switch from an AP to another.

### Data Analysis

The sociodemographic characteristics of the study population, as well as the characteristics of the recorded ADRs, were evaluated with a descriptive analysis. Mean and standard deviation (SD) values were calculated for the anthropometric values, laboratory tests, and cardiovascular examination results. Changes to continuous variables over time were analyzed using a linear mixed model in subsamples of our population, determined according to the AP received and presenting an adequate number of subjects to execute the analysis properly. The time factor for the model was set at baseline and months 6 and 12. Differences between the values recorded at the time points were considered significant if the associated *P* value was <0.05. For statistical analyses, the data processing software R: A Language and Environment for Statistical Computing, version 3.5.3, plus the Ime4 package, was used.

## Results

Starting from April 2017, 158 eligible patients who met the enrollment conditions entered into the study. Among these, 116 completed 12 months of monitoring. These patients had a mean ± SD age of 11.4 ± 3.5 years and were mainly of male sex (*n* = 82, 71%). Ninety were followed as outpatients, 20 were initially enrolled into the study as inpatients, and 6 have initiated the treatment while in day hospital service. Considering the primary diagnoses groups, the disruptive behavioral disorders (*n* = 39) was the most represented, followed by autistic spectrum disorders (*n* = 31) and psychotic disorders (*n* = 11) (see Table 1 for more details). In terms of *ICD-10*, the most frequently observed pathologies, alone or as a part of a more complex clinical picture, were attention-deficit hyperactivity disorders (code F90, *n* = 36), followed by autistic spectrum disorders (code F84, *n* = 35), conduct disorders (code F91, *n* = 20), and tic disorders (code F95, *n* = 19). Patients received for the most part (*n* = 104, 89.7%) an initial treatment with a single AP; however, a minority of patients (*n* = 12, 10.3%) were treated with two APs, of which half were initiated at the same time, whereas for the remaining half of subjects an add-on strategy was followed for the therapy. The APs used as first treatments were, in order, risperidone (*n* = 52, doses ranging from 0.25 to 7.5 mg), aripiprazole (*n* = 44, 2–20 mg), olanzapine (*n* = 7, 2–20 mg), haloperidol (*n* = 6, 0.4–4.8 mg), levomepromazine (*n* = 5, 37.5–75 mg), clozapine and quetiapine (both *n* = 4, 25–300 mg for clozapine and 25–250 mg for quetiapine), periciazine (*n* = 3, 0.5–4.5 mg), clotiapine (*n* = 2, 40–90 mg), and promazine (*n* = 1 with a dose of 90 mg). The evaluation of the impact of the AP administration over time on the anthropometrical measurements and laboratory and cardiologic parameters was carried out only on the subpopulations of patients being treated with risperidone (*n* = 52) and aripiprazole (*n* = 44), as those were the only ones with a sufficient number of subjects to perform the analysis correctly.

**Table 1:** Characteristics of patients who completed 12 months of treatment.

**Characteristics**	***n***	**%**
**Number of patients**	116	–
**SEX**		
Male	82	71
Female	34	29
**AGE GROUPS**		
13–18 y	47	41
7–12 y	56	48
0–6 y	13	11
**TYPE OF PATIENTS**		
Outpatients	90	78
Inpatients	20	17
Day hospital	6	5
**PRIMARY PSYCHIATRIC DIAGNOSIS (DSM-5)**		
Disruptive behavior disorders (conduct disorder, oppositional defiant disorder, attention-deficit/hyperactivity disorder)	39	34
Autistic spectrum disorders	31	27
Psychotic disorders (childhood schizophrenia, other psychotic disorders)	11	9
Tourette syndrome and tic disorders	7	6
Eating disorders (anorexia, bulimia)	6	5
Anxiety disorders (posttraumatic stress disorder, obsessive-compulsive disorder, generalized anxiety)	6	5
Cognitive impairments (with behavior disturbances)	6	5
Mood disorders (unipolar depression, bipolar disorder, dysthymia)	5	4
Personality disorders	3	3
Developmental disorders	1	1
Specific learning disabilities	1	1

The patients treated with risperidone were mainly of male sex (*n* = 40, 76.9%), and the observed mean ± SD age was of 9.7 ± 3.3 years. As far as the anthropometrical and metabolic parameters variations were concerned (Table 2), a significant increase in BMI was observed over time (*P* < 0.001). Also, the PRL concentration levels significantly increased during the study period (*P* = 0.04), whereas no significant differences were observed in values of fasting glucose, cholesterol, and triglycerides. In relation to the cardiologic examinations, a statistically significant increase was observed in the heart rate measurements values (*P* = 0.04). No statistically significant variations were observed in corrected QT (QTc) interval values. Nevertheless, two patients experienced prolongation of the QTc interval <450 ms, leading to treatment withdrawal. Stratifying the sample in two age groups (4–11 and 12–18 years), the observed increase in BMI and heart rate values remained significant in both age groups. Conversely, the increase in PRL concentrations levels remained significant only for the 12–18 year age group (*P* = 0.036) but not for the 4–11 year group (*P* = 0.066).

**Table 2:** Anthropometric and laboratory parameters variations in patients treated with risperidone.

**Parameter**	**Baseline**		**6 months**		**12 months**		**Over time variation**
	***n***	**Mean ± SD**	***n***	**Mean ± SD**	***n***	**Mean ± SD**	***p***
BMI (kg/cm^2^)	52	20.02 ± 3.71	52	21.05 ± 3.78	52	22.01 ± 3.55	<0.001
Fasting glucose (mg/dL)	52	85.55 ± 8.13	48	87.94 ± 8.92	50	88.48 ± 9.85	0.271
Total cholesterol (mg/dL)	52	155.95 ± 24.32	48	153.19 ± 27.28	50	164.68 ± 32.1	0.494
Triglycerides (mg/dL)	52	68.11 ± 41.46	48	64.83 ± 17.93	50	70.95 ± 21.03	0.154
Prolactin (ng/mL)	52	15.24 ± 9.37	47	26.58 ± 19.71	47	19.78 ± 13.94	0.04
Systolic pressure (mmHg)	52	97.35 ± 11.11	50	104.48 ± 15.17	51	101.61 ± 10.38	0.528
Diastolic pressure (mmHg)	52	59.22 ± 8.92	50	63.27 ± 7.86	51	64 ± 8.9	0.93
Heart rate (BPM)	52	83.92 ± 16.5	50	89.29 ± 16.55	51	87.11 ± 14.46	0.04
QT interval corrected	52	408.3 ± 22.74	46	403.1 ± 20.42	45	412 ± 20.24	0.695
White blood cells (μL)	52	8,385.67 ± 3,301.14	46	7,367.03 ± 2,144.29	47	7,362.22 ± 4,170.37	0.273
Neutrophil count (%)	52	48.73 ± 14.92	46	49.96 ± 10.99	47	52.11 ± 13.97	0.416

The aripiprazole group was mainly composed of male patients (*n* = 32, 72.7%) with a mean ± SD age of 11.9 ± 2.8 years. Considering the anthropometrical and metabolic parameters (Table 3), the BMI significantly increased over time (*P* < 0.001). A slight, but not statistically significant, decrease in PRL levels over time was observed. No significant differences were observed in fasting glucose, cholesterol, and triglycerides values during the study period. With regard to cardiologic examinations, the heart rate measurements showed a statistically significant increase (*P* = 0.026) over time. No statistically significant variations in QTc values were observed. The observed increase in BMI and heart rate values remained significant for both groups (4–11 and 12–18 years).

**Table 3:** Anthropometric and laboratory parameters variations in patients treated with Aripiprazole.

**Parameter**	**Baseline**		**6 months**		**12 months**		**Over time variation**
	***n***	**Mean ± SD**	***n***	**Mean ± SD**	***n***	**Mean ± SD**	***p***
BMI (kg/cm^2^)	44	20.02 ± 3.71	44	21.05 ± 3.78	44	22.01 ± 3.55	<0.001
Fasting glucose (mg/dL)	44	85.55 ± 8.13	41	87.94 ± 8.92	43	88.48 ± 9.85	0.402
Total cholesterol (mg/dL)	44	152.46 ± 20.47	41	155.87 ± 23.7	43	159.52 ± 24.72	0.125
Triglycerides (mg/dL)	44	76.69 ± 46.41	41	87.6 ± 66.42	43	72.52 ± 33.76	0.9
Prolactin (ng/mL)	44	9.57 ± 9.96	41	3.92 ± 4.66	39	2.63 ± 3.1	0.072
Systolic pressure (mmHg)	44	104.97 ± 11.23	42	104.75 ± 13.53	43	107.2 ± 13.72	0.107
Diastolic pressure (mmHg)	44	60.31 ± 7.55	42	63.15 ± 8.43	43	63.03 ± 8.54	0.704
Heart rate (BPM)	44	76.29 ± 13.76	42	82.11 ± 12.44	43	82.69 ± 12.97	0.026^a^
Qt interval corrected	44	402.76 ± 29.65	40	392.87 ± 43.67	38	402.01 ± 20.72	0.687
White blood cells (μL)	44	6,625.6 ± 1,721.33	39	6,167.22 ± 2,446.49	41	7,182.76 ± 3,194.2	0.882
Neutrophil count (%)	44	49.84 ± 8.77	39	52.98 ± 13.29	41	56.5 ± 10.23	0.223

a *The difference was statistically significant for baseline vs. 6 months and baseline vs. 12 months but not for 6 vs. 12 months*.

With regard to ADRs, 86 patients experienced at least one ADR, of which 61 were males (70.9%) and 25 (29.1%) were females, and 76 were treated with a single AP, whereas 10 were treated with 2 APs. Adverse drug reactions were most common with clozapine; all of the patients initially treated with this AP presented at least one ADR. Adverse drug reactions were also caused, in decreasing order, by levomepromazine (*n* = 5, 63%), aripiprazole (*n* = 36, 58%), risperidone (*n* = 39, 57%), olanzapine (*n* = 6, 50%), promazine (*n* = 1, 50%), quetiapine (*n* = 2, 40%), and haloperidol (*n* = 2, 29%). A total of 238 specific ADRs were observed (Table 4) with a mean of 2.8 reactions per patient. Reported ADRs were mostly (*n* = 213, 89.5%) classifiable as non-serious, and more than half of them showed a positive outcome (38.2% improved, and 17.6% fully recovered) (Table 5). Concerning serious ADRs (*n* = 25, 10.5%), only one required a prolongation of the hospitalization period for the patient, whereas the others were classified as other relevant conditions based on clinical judgment. Among these serious ADRs, those classifiable as psychiatric events (irritability, drowsiness, insomnia, tics, and visual hallucinations) were most common. A few other serious ADRs affected the cardiovascular (QTc interval prolongation, tachycardia, leukopenia, and increased arteriosus pressure), endocrine (creatine phosphokinase increased, hyperprolactinemia, amenorrhea), gastrointestinal (diarrhea, drooling, abdominal pain), metabolic (hypercholesterolemia,), and nervous systems (dystonia). Stratifying the ADRs by SOC, the most commonly reported one was “investigations” (*n* = 63, 26.5%) with PTs such as weight gain, increased serum PRL, QTc interval elongation, and blood creatine phosphokinase increased. Other frequent SOCs were “metabolism and nutrition disorders,” with terms such as hyperphagia, hypercholesterolemia, and hypertriglyceridemia (*n* = 39, 16.4%); “psychiatric disorders” including reactions such as insomnia, enuresis, irritability, and aggression (*n* = 39, 16.4%); nervous system disorders (*n* = 30, 12.6%), with terms like tremor, somnolence/sedation, headaches, and extrapyramidal disorder; and lastly, the SOC general disorders and administration site conditions, with PTs such as drug ineffective, condition worsened, and therapeutic response decreased (*n* = 24, 10.1%).

**Table 4:** Frequently observed ADRs stratified by PT.

**ADRs^a^**	***n***
Weight gain	34
increased Serum prolactin	21
Hyperphagia	20
Hypercholesterolemia	14
Drug ineffective	9
Enuresis	8
Tremor	8
Insomnia	8
Condition worsened	7
Somnolence	7
Reduced therapeutic response	6
Abdominal pain	4

a *ADRs observed <3 times have been excluded from the table*.

**Table 5:** Observed ADR characteristics.

	***n*^a^**	**%**
**SERIOUSNESS**		
Not serious	213	89.5
Serious—other clinically relevant condition	24	10.1
Serious—hospitalization	1	0.4
**OUTCOME**		
Improved	91	38.2
Not reported	56	23.6
Not yet resolved	49	20.6
Fully recovered	42	17.7
**SUSPECTED DRUGS^b^**		
Risperidone	102	38.1
Aripiprazole	96	35.8
Olanzapine	14	5.2
Clozapine	13	4.9
Quetiapine	17	6.3
Levomepromazine	14	5.2
Haloperidol	8	3
Promazine	4	1.5

a *The total number of adverse drug reactions (ADRs) (n = 238) exceeds the number of cases because several reports contained more than one ADR, and some patients experienced more than one of these several times*.

b *The ADR distribution stratified by drug is not mutually exclusive*.

## Discussion

Children and adolescents are not only small adults, but they are individuals in a state of dynamic physiological development that can be negatively impacted by ADRs (30). The use of APs in pediatrics is frequently long-term. However, the clinical evidence supporting the tolerability in these patients is derived from short-term studies that do not reflect the reality of common clinical practice accurately (19). For these reasons, we monitored pediatric patients with a follow-up period of 1 year. We evaluated the changes in the main clinical, cardiovascular, and laboratory parameters in accordance to the NICE guidelines and integrated these observations with a descriptive analysis of identified ADRs. Most of the patients who completed 12 months of continuous therapy with an AP were treated either with risperidone or aripiprazole. Therefore, the analysis was conducted on these subgroups.

### Monitoring of Parameters Over Time

Concerning anthropometric variables, we observed a statistically significant increase in BMI for both risperidone and aripiprazole treatment groups. This is in line with results from a previous open-label, randomized controlled study (31) and a number of retrospective observational investigations (32–34). A higher mean BMI was observed in patients treated with aripiprazole. This difference is likely due to the fact that risperidone is considered to cause more weight gain than aripiprazole. Indeed, this antipsychotic is prescribed with caution to children and adolescents presenting an elevated BMI prior to treatment (32). In addition to that, a placebo-controlled study on aripiprazole concluded that weight gain was more likely among young children already being overweight (35).

The increase in PRL levels is a common adverse effect that occurs when using both conventional and atypical APs (36). In patients treated with risperidone, a significant increase in PRL levels over time was observed, the highest mean value of which was observed at 6 months, and then the levels decreased between the 6th and 12th month of treatment. The mean PRL value, however, remained above the normality range (2–18 ng/mL) even at 12 months, and the difference between this value and the one recorded at baseline was still statistically significant. The lower levels of PRL observed at 12 months were more likely the result of preventive measures adopted by the clinicians such as dose reduction or addition of a dopamine agonist, rather than the product of a physiological adaptation to the treatment. Previous studies have also indicated the eventual development of tolerance to treatment as a factor contributing to the decrease of PRL values over time (37). Following the stratification of the sample in two age groups, the increase PRL levels remained significant only in patients in the 12–18-year age range. This is in agreement with a previous study indicating that adolescent patients seem to be more sensitive to increments in PRL levels after the initiation of a therapy with risperidone (38). Serum PRL increase with risperidone has been mostly asymptomatic in our sample. Indeed, of the 17 cases reported, only one was associated with clinical symptoms (amenorrhea). However, in 6 cases, a therapy discontinuation has been decided by the clinicians. In patients treated with aripiprazole, a slight and not statistically significant decrease in PRL values was observed during the observation period. In particular, three patients in the aripiprazole group had PRL level below the normal range (2–18 ng/mL). This is consistent with findings obtained by a previous investigation (31).

Our study has found no statistically significant differences in patients treated continuously for 1 year with risperidone or aripiprazole in relation to fasting glucose, total cholesterol, and triglycerides, as well as in white blood cell concentration and neutrophil percentage. These findings are in agreement with scientific literature (25, 31) and support the relative safety of these two APs in terms of these metabolic parameters.

Tachycardia is a well-known class effect for SGAs also reported in the Summaries of Product Characteristics of both risperidone- and aripiprazole-based formulations (39, 40). Our results show a slight but statistically significant increase in heart rate values for both treatment groups. The increase was more noticeable at 6 months, in line with previously reported literature (31). These changes in heart rate associated with the administration of APs are unlikely to be clinically significant. However, in our study population, one patient treated with risperidone experienced severe tachycardia and its clinical sequelae.

Concerning ECG evaluation, our study showed that the treatment with aripiprazole and risperidone was related with a mostly unmodified mean QTc. This study underlines the relative cardiac safety of aripiprazole and risperidone in children and adolescents, even after 1 year of therapy, as reported in previous investigations (41, 42). However, two of the enrolled patients treated with risperidone had a QTc exceeding 450 ms that led to the discontinuation of therapy before 12 months. This highlights the importance of monitoring QTc parameters during AP treatment in the pediatric population as indicated by NICE guidelines (26).

### Adverse Drug Reactions

Of the 158 initially enrolled patients, more than 54% experienced at least one ADR. The antipsychotic associated with the highest ADR incidence was clozapine. Indeed, the five patients who initiated treatment with clozapine developed at least one ADR. Several clinical trials in literature described significantly higher adverse event rates for clozapine when compared to other APs (43–45). Moreover, a recent study on the evaluation of clozapine use in pediatric patients pointed out a substantial rate of adverse events (46). However, the limited number of patients treated in the present study precludes us from drawing any conclusions in this sense. Among patients who experienced ADRs, 42% had more than one. In total, 238 specific ADRs have been observed. Most of the reported ADRs were already known for this class of drugs (e.g., weight gain, increased serum PRL, and hyperphagia), and most of them were classifiable as non-serious. Only 10.5% of the ADRs were either clinically relevant or, only in one case of an acute dystonia, required the patient to be hospitalized. No cases of life-threatening ADRs were observed. Among the serious ADRs, psychiatric effects have been the most represented ones (34.6%). Concerning these psychiatric ADRs, it is difficult to assign causality to APs with great certainty because of a potential worsening of the underlying condition. Only 56% of the ADRs showed a positive outcome (namely, an improvement in the 38.24% and a complete remission in the 17.7%). This is in line with what was reported in literature about long-term antipsychotic therapies that are often maintained without the appropriate safety measures (47). Although the ADRs recorded in our sample were mostly well-known effects, some of the long-term consequences of these ADRs on the neurodevelopment are still not entirely clear. One such example is the case of sustained levels of high or low PRL concentrations. Furthermore, even if classified as not serious, ADRs seem also to increase the rate of antipsychotic discontinuation (48–50).

### Strengths and Limitations

This is one of the few studies, in the context of pediatric psychiatry, to present real-world long-term monitoring data covering most of the parameters considered to be of clinical relevance by international guidelines for the monitoring of metabolic and cardiac tolerability, combined with a descriptive analysis of the ADRs recorded for the same population.

However, the results of our study should be interpreted keeping in mind its limitations, which are mainly related to its observational and naturalistic nature, such as the high variability within the sample, the lack of randomization and information on the drug naivety of the subjects included in the study. The relatively small sample size is also a limitation to consider. Despite our best efforts to recruit more patients, poor adherence to metabolic monitoring, and discontinuation of AP treatment limited enrollment. Our findings must be considered exploratory, and more conclusive evidence should be provided, in our opinion, using an interventional approach.

## Conclusions

This study provides information on the long-term tolerability of two of the most commonly used APs in the pediatric setting, combined with an overview of the tolerability and safety of APs in a naturalistic setting. The findings of this study confirm that risperidone and aripiprazole are relatively well-tolerated therapeutic options for the treatment of a variety of psychiatric disorders, even after 1 year of therapy. However, observational studies such as the present may suffer from selection bias, which tends to exclude patients with a lower tolerance or suboptimal compliance to the therapy (e.g., the two QTc prolongations discussed). Furthermore, findings such as the statistically significant relation between the exposure to these drugs, the increase in mean values of the BMI, the increase in heart rate values, and the variations over time in PRL levels for risperidone highlight the need for a systematic monitoring of the tolerability of APs in the pediatric population.

## Data Availability Statement

The datasets for this article are not publicly available because they contain sensitive data of pediatric patients. Requests to access the datasets should be directed to Edoardo Spina, espina@unime.it.

## Ethics Statement

The study was approved by the Ethical Committee of Messina.

## Author Contributions

ES: project coordination. GC, MB, CT, PVC, FV, FD, CR, AG, and EG: acquisition of data. GC, VS, and EG: analysis and interpretation of data. EG and ES: clinical evaluation of data. GC and EG: writing of the paper. PMC and ES: critical revision. ES: final approval of the version to be published. All authors listed have sufficiently made contributions to the entire content of the manuscript and have given their consent for publication.

### Conflict of Interest

The authors declare that the research was conducted in the absence of any commercial or financial relationships that could be construed as a potential conflict of interest. The reviewer CP declared a past co-authorship with one of the authors AG.
